# Beyond BMI: a comprehensive approach to pediatric obesity assessment

**DOI:** 10.3389/fped.2025.1597309

**Published:** 2025-05-22

**Authors:** Norma Cipatli Ayuzo Del Valle, Perla Pérez-Treviño, Ana Carla Cepeda Lopez, Regina M. Murillo-Torres, Elena Cristina Castillo, Diego Gutierrez-Cantu, Oscar Tamez-Rivera, Mayela Paez Flores, Isabella Flores-Ayuzo, Luis Alberto Luévano-Martinez, Sergio Javier Fernandez Ortiz, Noemí García, Leonardo Mancillas-Adame

**Affiliations:** ^1^ Tecnologico de Monterrey, Escuela de Medicina y Ciencias de la Salud, Monterrey, NL, Mexico; ^2^ Tecnologico de Monterrey, The Institute for Obesity Research, Monterrey, NL, Mexico; ^3^ Department of Nutrition, School of Mathematics, Science and Engineering, University of the Incarnate Word, San Antonio, TX, United States; ^4^ Tecnologico de Monterrey, Instituto de Pediatria, Monterrey, NL, Mexico

**Keywords:** pediatric obesity, body mass index, metabolic health, childhood nutrition, physical activity, body composition

## Abstract

**Background:**

Traditional Body Mass Index based obesity classification presents limitations in pediatric populations, particularly among physically active children. The 2025 Obesity Classification Framework proposed by The Lancet Diabetes & Endocrinology Commission integrates body fat distribution and metabolic biomarkers, aiming to enhance diagnostic accuracy in pediatric obesity.

**Methods:**

We evaluated 111 physically active children (aged 5–11 years) from the Monterrey Football League in Mexico using both the traditional BMI-based classification and the new 2025 Obesity Classification Framework, which incorporates body composition (measured by bioelectrical impedance analysis), waist-to-height ratio, and metabolic biomarkers. Each participant was classified with both frameworks, and outcomes were compared against metabolic risk markers. Normality was assessed using the Shapiro–Wilk test. Non-normally distributed variables (fat mass, visceral fat, triglycerides, creatinine, and pCr) were analyzed using non-parametric tests, while parametric tests were applied for normally distributed data. Agreement between classifications was determined using Cohen's kappa coefficient.

**Results:**

Agreement between classifications was moderate (*κ* = 0.532, *P* < 0.001). Using the new framework, 20 children previously classified as overweight by BMI were reclassified as having preclinical obesity, reflecting excess adiposity previously unrecognized. Conversely, four participants initially categorized as obese by BMI were reclassified as non-obese, reflecting elevated lean mass rather than adiposity. Participants categorized as having preclinical obesity exhibited significantly higher levels of LDL cholesterol and apolipoprotein B compared to non-obese peers.

**Conclusions:**

The 2025 Obesity Classification Framework provides greater precision than traditional BMI-based assessments by effectively differentiating between excess adiposity and increased lean mass in physically active children. Although bioelectrical impedance analysis was selected due to its practicality, cost-effectiveness, and non-invasiveness, it has inherent measurement variability compared to dual-energy x-ray absorptiometry. Future research validating these results against DXA or other reference standards is recommended. Adopting this comprehensive assessment strategy may facilitate earlier and more targeted interventions for children at risk of obesity-related complications.

**Clinical Trial Registration:**

https://doi.org/10.1186/ISRCTN12172320, identifier ISRCTN12172320.

## 1 Introduction

Pediatric obesity is one of the most significant public health challenges of the 21st century, with far-reaching consequences for individual health and healthcare systems worldwide (1). Obesity is often defined as excess body weight relative to height, but this simplistic definition fails to capture its complex etiology. Body Mass Index (BMI) is a widely used, low-cost tool, defined as weight in kilograms divided by height in meters squared, and has served its purpose in providing a simple measure of obesity. However, it fails to capture the full complexity of obesity, which is primarily characterized by excess body fat rather than overall body size. Since obesity can have varying impacts on metabolism and health, BMI falls short in reflecting the diverse factors that contribute to the condition. This limitation becomes particularly pronounced when assessing children. As they grow, their body composition changes significantly, influenced by factors such as age, sex, pubertal status, and physical activity levels (2). These variables can cause BMI to misclassify the health status of children, highlighting the need for more comprehensive and nuanced methods to assess obesity.

Recognizing these limitations, The Lancet Diabetes & Endocrinology Commission introduced a new, evidence-based framework in January 2025 (2025-OCF), to provide a more precise classification of obesity (3). This model distinguishes between two key stages: (1) Preclinical Obesity: A stage characterized by the presence of excess adiposity that has not yet resulted in measurable organ dysfunction or significant impairment in daily activities. While these children may appear asymptomatic, they are at heightened risk for developing metabolic disorders, cardiovascular disease, and other obesity-related conditions later in life (3, 4). (2) Clinical Obesity: A chronic, systemic disease where excess adiposity has already led to physiological damage or functional limitations. This includes early-onset type 2 diabetes, hypertension, dyslipidemia, hepatic steatosis, orthopedic complications, and respiratory disorders, all of which are increasingly observed in pediatric populations (3–5).

The 2025-OCF has been proposed as a more precise approach to obesity classification by incorporating body composition and metabolic markers. However, it has not yet been validated in pediatric populations, where obesity presents unique physiological and metabolic characteristics distinct from adults. Given the limitations of BMI in children, particularly among those with high levels of physical activity, validating this framework is essential to determine its accuracy and clinical relevance. Additionally, it should be tailored to specific populations to ensure accurate and meaningful application across diverse groups (6).

Pediatric obesity is a strong predictor of lifelong health outcomes. Children with clinical obesity are significantly more likely to experience persistent metabolic dysregulation, cardiovascular disease, and reduced life expectancy (7). Furthermore, obesity is linked to psychological and neurodevelopmental challenges, including increased risks of depression, anxiety, and disordered eating behaviors (8). Given these long-term consequences, it is essential to evaluate the utility of early identification strategies and targeted interventions. Assessing their impact can help determine their role in preventing the progression from preclinical to clinical obesity and in shaping appropriate approaches to address obesity-related health risks in adulthood.

Childhood obesity is a growing health concern worldwide, with severe consequences for long-term metabolic and cardiovascular health. While Mexico has one of the highest rates of childhood obesity, affecting nearly one in three children, this is not an issue exclusive to a single country (9). Many nations, including the United States, Brazil, India, and several European countries, are facing similarly high and rising rates of childhood obesity (10). The prevalence of metabolic syndrome, insulin resistance, and type 2 diabetes in children from these regions further underscores the need for a more precise and individualized obesity classification (11).

The framework proposed offers potential as a useful tool, especially in countries where socioeconomic and cultural factors heavily influence dietary habits, physical activity levels, and access to healthcare (12). Moreover, this framework could be valuable for the evaluation of body composition in physically active children, especially those participating in sports where weight categories influence classification, training strategies, and competition eligibility (13–15). By incorporating a refined diagnostic methodology, this classification system may aid in assessing early intervention strategies, identifying potential misclassification issues, and exploring its impact on long-term health outcomes on a global scale.

The objective of this study is to apply the 2025-OCF in a prospective study and assess whether it results in different categorizations compared to traditional BMI-based methods. By integrating additional body composition measurements, such as fat percentage and metabolic markers, this study seeks to explore whether this approach offers an alternative perspective on obesity classification in children aged 5–11 years who play American football. Additionally, this study will assess whether the new classification framework improves differentiation between excess adiposity and increased muscle mass in young athletes, a distinction that BMI alone may fail to capture (16). Ultimately, the findings will provide insights into the practical applicability of this new diagnostic approach and determine whether it offers advantages in identifying obesity risk while avoiding the misclassification of physically active children, which could otherwise lead to unnecessary dietary interventions and increased risk for early-onset eating disorders (8).

## 2 Materials and methods

### 2.1 Study design

A cross-sectional study was conducted from September to December 2024 among young athletes in northern Mexico to evaluate anthropometric and body composition measurements, as well as clinical and biochemical parameters to compare the 2025-OCF with the traditional BMI-based method for obesity classification in children. The study protocol was designed and conducted according to the ethical principles outlined by the 1964 Declaration of Helsinki and its subsequent amendments. Approval was obtained from the Research Ethics Committee of Hospital La Misión (registration number: HZHRMA-tE01-001). The study was prospectively registered in the ISRCTN clinical trial registry (ID ISRCTN12172320). All assessments were performed at Zambrano Hellion Hospital, Tecnologico de Monterrey, Mexico, following the ethical and institutional requirements.

### 2.2 Study population

Athletes aged 5–11 years were recruited from the Monterrey Football League (MFL), Mexico, (All participants were of Latino origin, reflecting the demographic characteristics of the study setting in northern Mexico; no other ethnic groups were excluded, but were not represented in this cohort). Inclusion criteria comprised Latino children aged 5–11 years enrolled in MFL, with at least one year of continuous participation and involvement in the previous season. All participants trained 2 h daily, Monday to Friday, and played matches on weekends, completing 12 h of structured physical activity weekly under standardized, attendance-controlled programs.

Exclusion criteria included conditions affecting metabolism or body composition, such as hypothyroidism, short stature requiring growth hormone therapy, type 1 diabetes, autoimmune diseases, or use of medications known to influence metabolic parameters (e.g., metformin, chronic corticosteroids). Written informed consent and assent were obtained from each participant and their parent or guardian before enrollment. Medical history was collected through structured, in-person interviews conducted by trained medical personnel.

### 2.3 Anthropometric and body composition measurements

Anthropometric and body composition measurements were performed by a trained pediatric physician following standardized protocols to minimize variability. Height was measured using a calibrated stadiometer (InKids, InLabs50, precision 0.1 cm, Seoul, Korea), which was calibrated using a Seca 213 Portable Stadiometer (Hamburg, Germany: Seca GmbH & Co. KG). Measurements were taken with the child standing barefoot, feet together, heels, buttocks, and upper back touching the vertical surface, and head positioned in the Frankfurt plane. Body composition parameters, including body weight, body fat percentage, skeletal muscle mass (kg), fat mass (kg), waist-to-hip ratio (WHR), and visceral fat index, were assessed using bioelectrical impedance analysis (BIA; InBody 120, InBody Co., Ltd., Seoul, Korea), BIA measurements were calibrated according to the manufacturer's standard guidelines for clinical research purposes. To ensure accuracy, BIA was conducted in the early morning before training sessions. Participants were instructed to avoid intense physical activity for 24 h prior to the measurement, to arrive in a fasting state, and to void their bladder before assessment. During the BIA procedure, they were asked to stand upright with arms and legs slightly apart to prevent contact between limbs and ensure accurate impedance readings.

BMI was assessed using WHO growth charts, classifying participants with Overweight (≥85th and <95th percentile) or Obesity (≥95th percentile) according to age- and sex-specific standards. Given ethnic differences in fat distribution and metabolic risk, body fat percentage thresholds from the FUPRECOL study (6) (validated for Latino children) were used to define excess adiposity (+2 SD from the mean for age- and sex-specific reference values). The thresholds were as follows: Ages 6–9 years: Males ≥34.5%, Females ≥33.0%; Ages 9–10 years: Males ≥34.5%, Females ≥33.5%; Ages 10–11 years: Males ≥34.0%, Females ≥35.8%; Ages >11 years: Males ≥31.7%, Females ≥35.6%. WHR was also used as an anthropometric obesity criterion, defined as WHR exceeding +2 SD for age- and sex-specific reference values, based on WHO growth standards. All anthropometric and body composition values were adjusted for age and sex to ensure accurate comparisons.

### 2.4 Biochemical analyses

Participants fasted for 12 h before blood sample collection. A total of 5 ml of venous blood was drawn into BD Vacutainer tubes without anticoagulant for serum collection. Biochemical analyses included total cholesterol, high-density lipoprotein cholesterol (HDL-C), low-density lipoprotein cholesterol (LDL-C), triglycerides, and fasting glucose. Altered laboratory parameters, were defined as: Fasting glucose >100 mg/dl; Total cholesterol >170 mg/dl; LDL cholesterol >130 mg/dl; Triglycerides >100 mg/dl (<10 years) or >130 mg/dl (≥10 years) and Apolipoprotein B > 90th percentile for age and sex. All tests were conducted using standardized enzymatic colorimetric methods and analyzed via spectrophotometry at the clinical laboratory of Zambrano Hellion Hospital, TecSalud, Tecnológico de Monterrey, Mexico.

### 2.5 Obesity classification using the 2025 obesity classification framework: preclinical and clinical assessment

To complement BMI-based classification, the 2025-OCF was applied, incorporating body composition analysis and metabolic markers to enhance obesity assessment. Under this framework: Preclinical obesity was defined as excess adiposity, confirmed by elevated body fat or a WHR exceeding +2 SD and an elevated body fat percentage, regardless of BMI. Clinical obesity was diagnosed when excess adiposity was accompanied by at least one of the following altered laboratory parameters such as fasting glucose, total cholesterol, LDL cholesterol, triglycerides, and apolipoprotein B.

### 2.6 Clinical manifestations

Functional limitations were assessed based on key clinical symptoms described in the pediatric section of the 2025-OCF (6), including respiratory (exercise-induced dyspnea, sleep apnea), orthopedic (hip slip, flat feet, genu varum or knee issues), renal (nocturnal enuresis), and neurological (headache) symptoms. These were evaluated through structured interviews with participants and caregivers, focusing on the presence or absence of these conditions. Some conditions listed in the classification, such as diabetes and other chronic diseases, were exclusion criteria, as previously described.

### 2.7 Data analysis

All statistical analyses were conducted using IBM SPSS Statistics (30.0.0-2024). The normality of body composition and biochemical variables was assessed using the Shapiro–Wilk test. Non-normally distributed variables, including body fat mass (kg), visceral fat, and triglycerides, were analyzed with nonparametric statistical methods, while normally distributed variables were examined using parametric tests. Demographic variables were analyzed using descriptive statistics, with continuous variables reported as means ± standard deviations (for normally distributed data) or medians and interquartile ranges (for non-normally distributed data). To evaluate the agreement between traditional BMI-based classification and the 2025-OCF, the kappa coefficient (*κ*) was calculated.

## 3 Results

**Figure 1:**
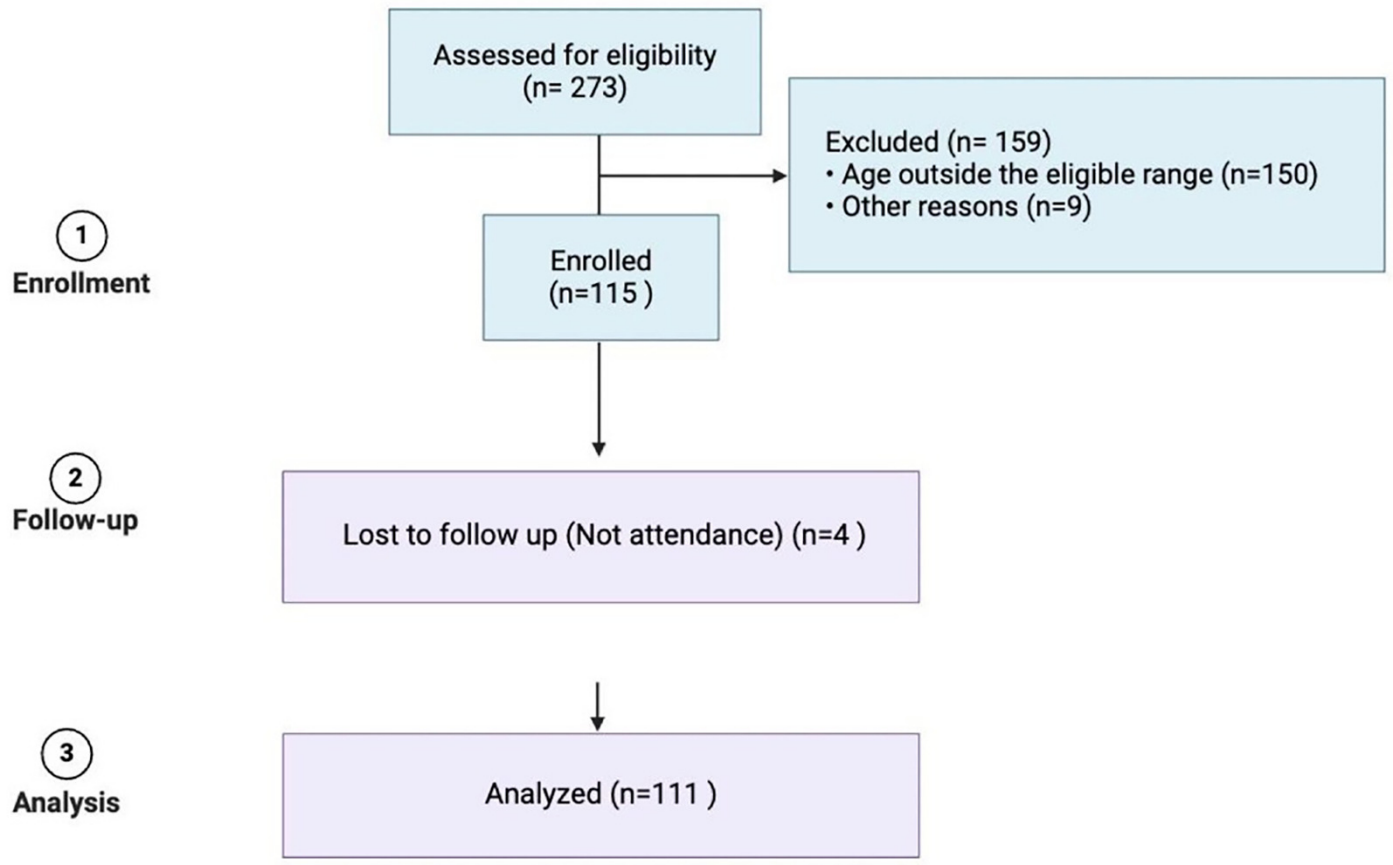
Patient-recruitment flow chart.

A total of 273 families contacted the study call center; however, only 115 children met the inclusion criteria, with the most common reason for exclusion being an ineligible age range. Ultimately, 111 participants were included in the final analysis, as 4 individuals missed their scheduled appointments (Figure 1).

### 3.1 Demographic and anthropometric characteristics

**Figure 2:**
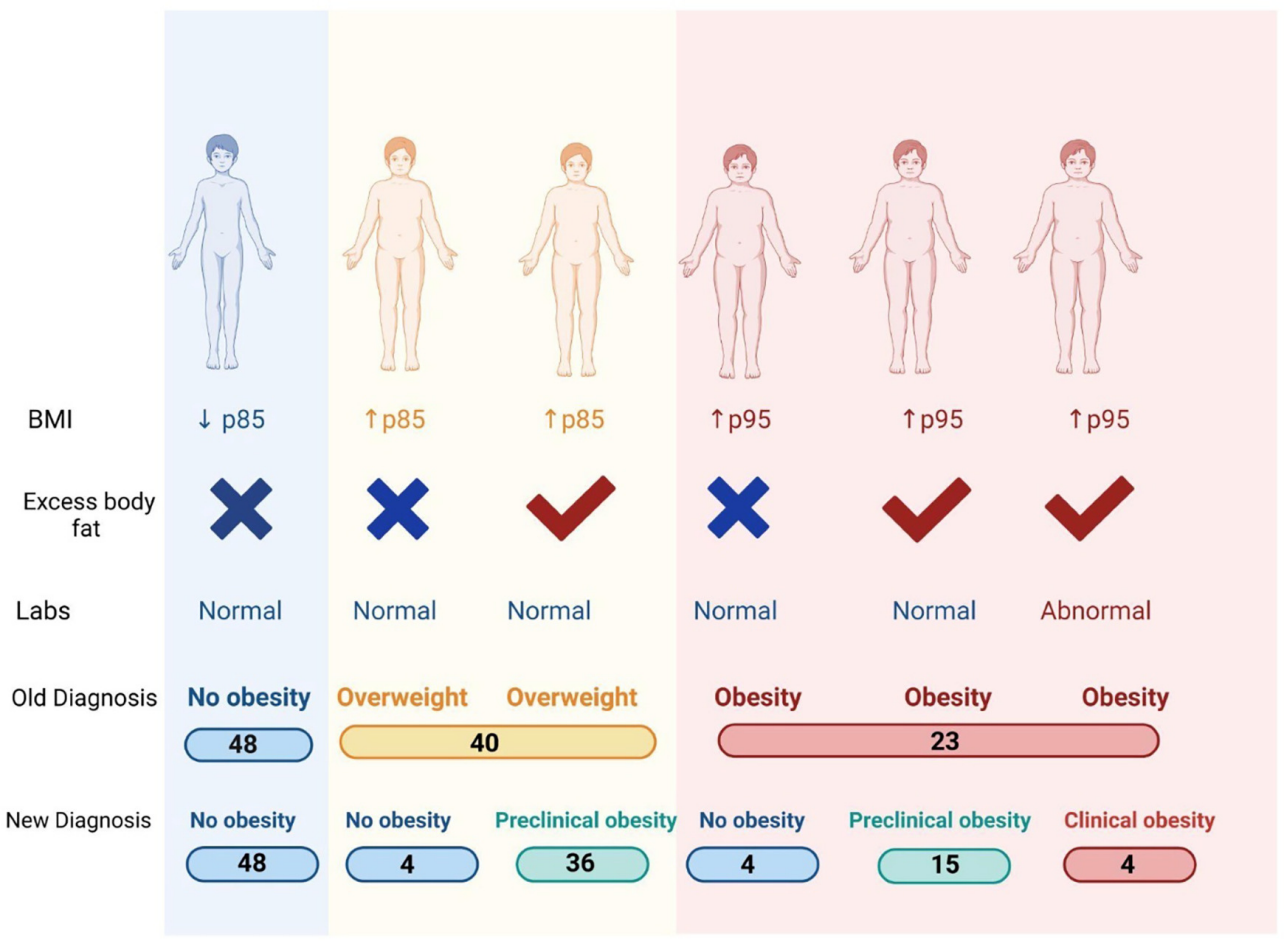
Distribution of obesity prevalence according to different classification methods: non-obesity, overweight, preclinical obesity, and clinical obesity, represented for each method.

A total of 111 participants, aged 6–11 years, were included in the study. The mean age was 10.0 ± 1.6 years. Among them, 74.1% (*n* = 83) were male and 25.9% (*n* = 28) were female. Based on traditional BMI criteria (WHO growth charts), 88 children (79.3%) were classified as non-obese, while 23 (20.7%) were categorized as obese. Notably, among the non-obese participants, 40 children (36.0%) fell into the overweight category. When classified by body fat percentage, 41 participants (36.9%) were classified as obese, while 70 (63.1%) were classified as non-obese. Using the 2025-OCF classification, 4 children were classified with obesity (3.60%), 51 children with preclinical obesity (45.95%), and 56 children without obesity (50.45%).The distribution of these classifications is illustrated in Figure 2. The agreement between BMI-based classification and body fat percentage classification was assessed using Cohen's kappa coefficient (*κ* = 0.532, *P* < 0.001), indicating a moderate level of concordance between the two methods. Sex-based differences were observed in body composition and metabolic parameters.

Among the 23 participants with a WHR above +2 SD, none were classified as “no obesity” by the 2025 Obesity Classification Framework (OCF). Of these, 19 were categorized as having preclinical obesity and 4 as clinical obesity, indicating a 100% overlap with OCF-defined risk categories. This suggests that WHR alone can effectively identify children at risk according to the OCF. However, WHR was not sufficient to distinguish between preclinical and clinical obesity, which requires the integration of metabolic and clinical criteria.

Excess adiposity was more prevalent in males than females (*p* = 0.03). No significant differences were found in ApoB or LDL cholesterol (*p* > 0.64), but triglycerides were significantly higher in males (103.05 mg/dl vs. 86.48 mg/dl, *p* = 0.002). We analyzed the relationships between lipid variables (HDL cholesterol, triglycerides, and total cholesterol) and obesity status using Pearson's correlation for HDL and total cholesterol, and Spearman's rank correlation for triglycerides due to its skewed distribution. Partial correlation analyses were performed to adjust for age and sex. HDL cholesterol showed a significant negative correlation with BMI-for-age (Pearson *r* = –0.35, *p* < 0.001) and body fat percentage (*r* = –0.40, *p* < 0.001). Triglycerides were positively correlated with BMI-for-age (Spearman *ρ* = 0.30, *p* = 0.002) and body fat percentage (*ρ* = 0.35, *p* < 0.001). Total cholesterol exhibited weaker but significant correlations with BMI-for-age (*r* ≈ 0.20, *p* = 0.03) and body fat percentage (*r* ≈ 0.22, *p* = 0.02), suggesting a modest association with adiposity measures. WHR above +2 SD for age and sex was observed in 23 participants (20.7%), indicating central obesity. WHR showed a strong correlation with visceral fat percentage (Pearson *r* = 1.0, *p* < 0.001), reinforcing its role as an indicator of adiposity distribution. Significant differences (*p* < 0.0001) were observed across BMI, body fat percentage, skeletal muscle mass (kg), waist-to-hip ratio, and basal metabolic rate (BMR) between non-obese and obesity groups. These findings were consistent regardless of classification method. The full comparison of anthropometric and body composition parameters is presented in Table 1. Importantly, four participants with high skeletal muscle mass (>90th and 95th percentile) were misclassified as obese by BMI but had normal visceral fat and WHR, highlighting the importance of integrating body composition analysis in obesity classification.

**Table 1:** BMI and body fat classification.

Variable	BMI – non-obesity	BMI – obesity	*p*-value (BMI)	Body fat (%) – non-obesity	Body fat (%) – obesity	*p*-value (Body fat)
*N* (Male; Female)	88 (68; 20)	23 (14; 9)	–	70 (53; 17)	41 (29; 12)	–
Age (years)	9.35 ± 1.6	9.26 ± 1.5	0.680	9.3 ± 1.6	9.3 ± 1.5	0.132
BMI (kg/m^2^)	18.9 ± 3.7	27.3 ± 4.3	**<0.001**	17.7 ± 2.8	25.8 ± 3.9	**<0.001**
Body Fat (%)	24.4 ± 8.7	41.3 ± 5.6	**<0.001**	21.0 ± 6.2	39.7 ± 4.6	**<0.001**
Visceral Fat	3.0 (2.0–6.0)	13.0 (8.0–16.0)	**<0.001**	3.0 (2.0–6.0)	13.0 (8.0–16.0)	**<0.001**
Muscle (kg)	14.5 ± 4.2	17.9 ± 4.7	**0.001**	14.0 ± 4.2	17.3 ± 4.3	**<0.001**
WHR	0.78 ± 0.06	0.91 ± 0.05	**<0.001**	0.77 ± 0.05	0.89 ± 0.05	**<0.001**

BMI, body mass index; WHR, waist to hip ratio.

**Bold values highlights statistically significant *p*-values.

### 3.2 Biochemical parameters and clinical manifestations

**Figure 3:**
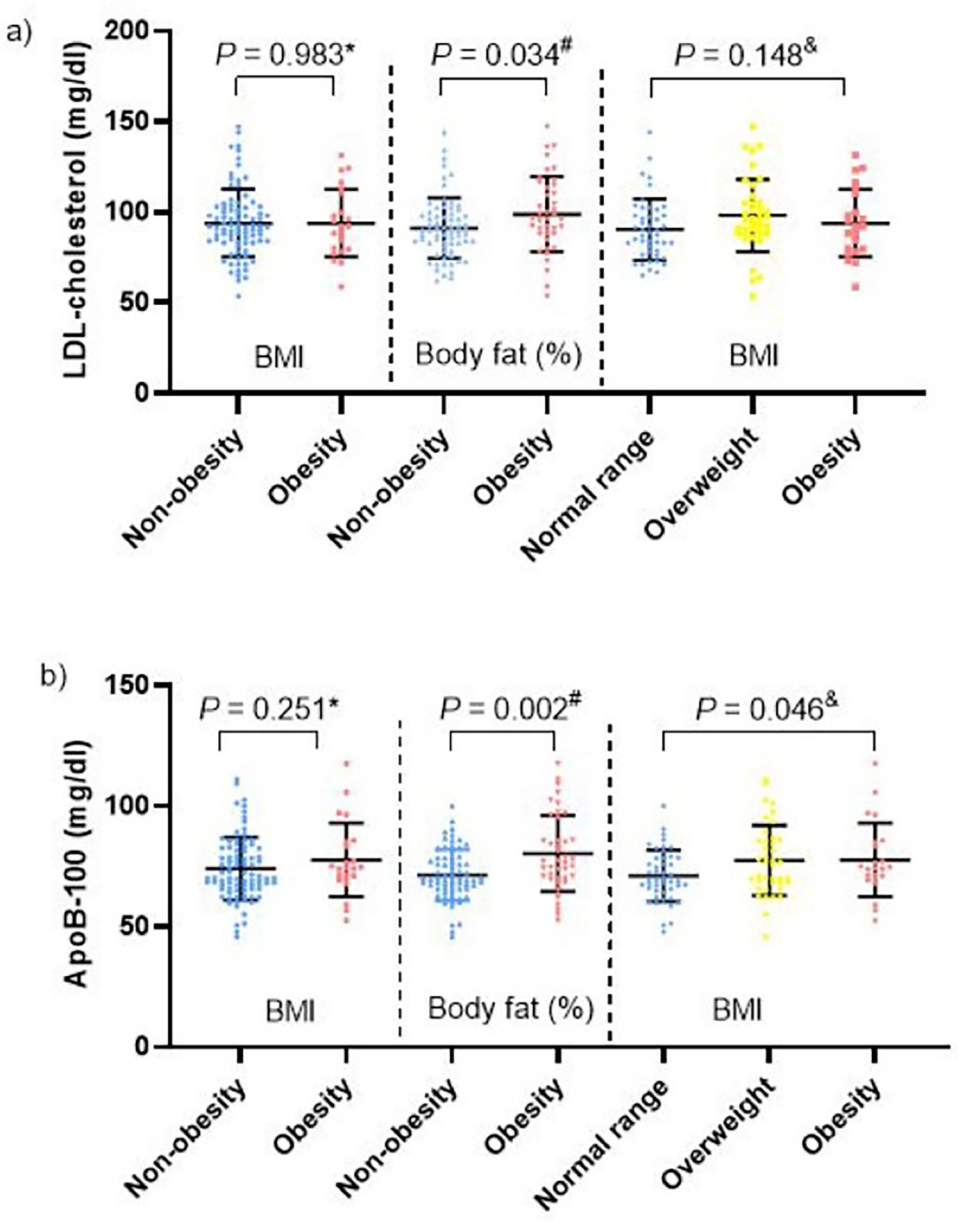
Correspondence of obesity classifications in comparison with **(a)** LDL-cholesterol and **(b)** ApoB. Mann–Whitney *U* Test, Student's *t*-test for independent samples, One-way ANOVA.

The classification based on body fat percentage proved more effective in identifying alterations in LDL cholesterol and apolipoprotein B (ApoB) compared to BMI-based classification. LDL cholesterol was significantly higher in the elevated body fat percentage group (98.8 ± 20.7 mg/dl) compared to the normal body fat percentage group (91.0 ± 16.7 mg/dl).In contrast, BMI-based classification failed to detect significant differences in LDL cholesterol, whether using two groups (obese vs. non-obese) or three groups (obese, overweight, and normal weight) (Figure 3A). Additionally, ApoB levels were also significantly higher in children with the high adiposity (80.3 ± 15.8 mg/dl) compared to the lean group (71.4 ± 10.6 mg/dl) (Figure 3B). Again, BMI-based classification did not distinguish significant differences in ApoB levels between groups. In contrast, glucose and triglyceride levels showed no significant differences, regardless of classification method. Notably, despite a substantial proportion of participants being classified with obesity, only a small fraction exhibited metabolic abnormalities: One participant had elevated fasting glucose (111 mg/dl). In follow-up, this patient was diagnosed with fasting glucose intolerance and was classified as obese by both classification methods. Another participant presented elevated triglycerides, high ApoB, and an elevated WHR and only four children reported physical symptoms, including moderate exertional dyspnea, orthopedic issues affecting the hip or knee, and ongoing evaluation for suspected sleep apnea.

### 3.3 Comparison between traditional BMI classification and the 2025-OCF

**Figure 4:**
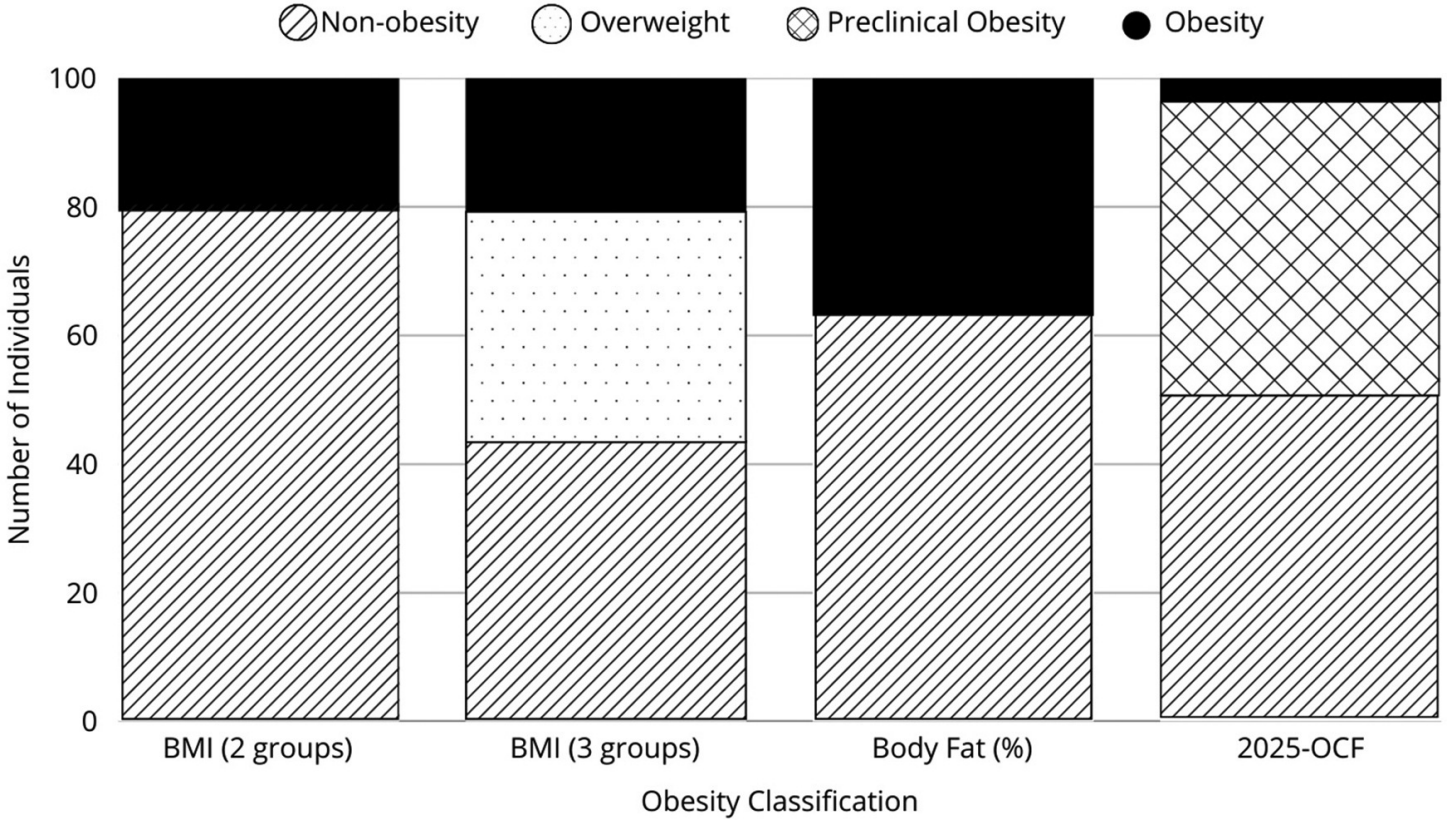
Classification of pediatric athletes: comparison between traditional BMI classification and the 2025-OCF. A total of 48 participants remained classified as normal weight. Four participants with a BMI ≥85th percentile were reclassified as non-obese because they did not meet criteria for excess body fat or increased waist-to-hip ratio (WHR). Thirty-six participants with a BMI ≥85th percentile had both excess body fat and increased WHR, placing them in the preclinical obesity category. Four participants with a BMI ≥95th percentile were reclassified as non-obese, as their high BMI resulted from increased skeletal muscle mass (above the 90th or 95th percentile) rather than excess adiposity or WHR elevation. Fifteen participants with a BMI ≥95th percentile, excess body fat, and increased WHR but no metabolic abnormalities were classified as preclinical obesity, bringing the total to 19 preclinical obesity cases when combined with those classified based on body fat percentage alone. Only four participants met the criteria for clinical obesity, having a BMI ≥95th percentile, excess body fat, increased WHR, and abnormal metabolic markers, including elevated fasting glucose, cholesterol, or ApoB.

The 2025 Obesity Classification Framework provided a more complete categorization of participants, differentiating between normal weight, preclinical obesity, and clinical obesity by incorporating waist-to-hip ratio (WHR), clinical symptoms, adiposity, and metabolic markers (Figure 4).

## 4 Discussion

Under the traditional BMI-based classification, 23 children were categorized as obese, while 40 were classified as overweight. However, the 2025 classification removes the term “overweight” and introduces the category of preclinical obesity, which identifies patients with excess adiposity without metabolic alterations. As a result, we found that a significant number of children who were previously classified as overweight using BMI were reclassified. In total, the new classification identified 19 cases of preclinical obesity and 4 cases of clinical obesity. Additionally, some children previously categorized as overweight or with obesity by BMI, were reclassified as normal weight under the new OFC. This reclassification offers a more comprehensive and accurate evaluation of obesity, leading to a more precise assessment of adiposity and metabolic risk. Despite their high levels of physical activity, the 2025-OCF identified a larger subset of children requiring closer monitoring, reinforcing the need for a classification system that integrates body composition, clinical symptoms and metabolic markers, rather than relying solely on BMI percentiles (17).

In our study, four children classified as obese based on BMI had high muscle mass without excess body fat, increased WHR, or altered biochemical parameters. It is well established that the inclusion of young athletes, who typically exhibit a higher muscle-to-fat ratio, may lead to an overestimation of lean mass and an underestimation of true adiposity when using BMI alone (18). Nonetheless, this athletic profile provides valuable insights into the limitations of BMI in active populations and underscores the need to incorporate additional body composition measures, such as visceral fat and metabolic markers, to achieve a more accurate assessment of obesity. Our findings indicate that body fat percentage is more strongly associated with lipid alterations than BMI-for-age, particularly in the case of HDL cholesterol and triglycerides, which showed stronger correlations with body fat percentage than with BMI. This suggests that body fat percentage may better reflect metabolic risk in pediatric athletes. Given that BMI does not distinguish between lean and fat mass, relying solely on BMI-for-age may underestimate metabolic alterations in children with higher muscle mass. By distinguishing true adiposity from increased skeletal muscle mass, the OCF eliminates the obesity misclassification found in the BMI-based classification, ensuring a more accurate and clinically meaningful assessment of pediatric obesity in young athletes (19). Incorrectly labeling young athletes as obese could lead to inappropriate dietary restrictions, negatively impacting growth, performance, and psychological well-being (20). Evidence suggests that early exposure to restrictive dieting increases the risk of disordered eating behaviors, emphasizing the importance of accurate obesity classification to prevent unnecessary dietary interventions (20, 21). However, the introduction of the “preclinical obesity” category raises additional concerns regarding its psychological and social implications. Labeling a child with preclinical obesity, may influence parental perception of their child's health, potentially leading to heightened anxiety, stigmatization, or unnecessary lifestyle modifications. Parental concern regarding a child's weight status has been associated with an increased risk of body dissatisfaction and maladaptive eating behaviors in children (22). Therefore, while this framework aims to improve obesity assessment, its implementation must consider the potential unintended consequences of such classifications on children's self-perception, family dynamics, and long-term health behaviors.

In our study, we observed some sex-based differences in adiposity and metabolic parameters. Males had a higher prevalence of excess adiposity according to FUPRECOL thresholds (6), while triglyceride levels were significantly higher in males than in females (*p* = 0.002). This may be related to known physiological differences, as pediatric studies suggest that males tend to accumulate more visceral fat, whereas females have a higher proportion of subcutaneous fat (7, 14). These differences could also be influenced by hormonal variations, with testosterone favoring lean mass development in males and estrogen progressively increasing adiposity in females during puberty (14). Additionally, previous research has reported that males may exhibit a less favorable lipid profile in the presence of obesity (11). However, the unequal sample size (92 males vs. 20 females) should be considered when interpreting these findings.

Another significant limitation of this study is that insulin was not measured, which prevented the calculation of the HOMA index and a direct evaluation of insulin resistance. However, it is important to note that physiological insulin resistance is expected during puberty, and measuring insulin levels could introduce confounding bias (22). Furthermore, while BIA was selected for assessing body composition due to its non-invasive nature and cost-effectiveness (23), it is important to recognize its limitations compared to more precise techniques, such as dual-energy x-ray absorptiometry (DXA) (22). Although BIA offers a practical and accessible method for estimating body composition, its inherent variability in measuring fat mass and lean mass remains a noteworthy drawback (23–25). This is particularly relevant in pediatric athletes, where the precision of body composition measurements is critical. Consequently, despite the advantages of BIA in routine clinical evaluations, future research should consider validating these findings using DXA or other gold-standard methodologies to further substantiate the reliability of the new classification framework and enhance its applicability in diverse clinical settings (26).

Additionally, WHR is another valuable anthropometric tool included in the 2025 framework.While WHR identified a similar proportion of participants as BMI-based classification, the two measures were not fully concordant, highlighting that WHR may serve as a complementary, cost-effective tool to detect central adiposity and refine obesity risk assessment. WHR is a simple and cost-effective alternative that only requires a measuring tape, making it an accessible option for populations with limited access to advanced technologies (19). WHR has long been recognized as a significant indicator of body fat distribution and is a useful measure for assessing visceral adiposity, studies have demonstrated its predictive ability for identifying adiposity and metabolic risk in pediatric populations (27). Given its ease of use, WHR represents a practical alternative for assessing obesity classification, particularly in large-scale epidemiological studies or resource-limited settings. Furthermore, WHR can more accurately reflect the risk of obesity-related health issues, particularly in individuals with a normal BMI but high abdominal fat. In the 2025-OCF, WHR has been described as an effective tool for improving nutritional assessments in pediatric patients, providing a feasible method for addressing obesity classification in diverse populations, offering a more comprehensive and accurate evaluation of obesity and metabolic risk. While WHR demonstrated perfect overlap with OCF-defined obesity risk categories, it lacks the capacity to distinguish between preclinical and clinical obesity, underscoring its value as a screening tool rather than a stand-alone diagnostic method.

Special consideration should be given when applying this classification framework to children undergoing puberty. During this developmental stage, a temporary increase in body fat percentage is often necessary before the pubertal growth spurt (14). As such, classifying a child as having preclinical obesity should serve as a guideline for monitoring, rather than an automatic indication of poor nutritional status (24). Some studies suggest that maintaining higher BMI levels may even be associated with certain longevity benefits in specific populations (28–30). While the 2025-OCF enhances diagnostic accuracy, its implementation may pose challenges related to accessibility, cost, and integration into routine pediatric assessments, particularly in resource-limited settings.

The 2025-OCF (3) presents challenges in defining diagnostic criteria for pediatric populations. While the initial classification steps (BMI assessment followed by body composition measures such as body fat percentage or waist-to-hip ratio) are relatively straightforward, determining preclinical obesity vs. clinical obesity remains complex. Although laboratory markers with established cutoff values exist, their application in children requires careful consideration. The distinction between preclinical and clinical obesity remains conceptually and diagnostically complex in pediatric populations. While the 2025 Obesity Classification Framework provides a structured, stepwise approach to stratify obesity-related risk, it acknowledges that the progression from adiposity to metabolic dysfunction is gradual and influenced by multiple biological and environmental factors (3). This continuum challenges binary definitions and underscores the need for close clinical monitoring rather than rigid labeling, especially in children who may not yet exhibit overt metabolic alterations (4). Therefore, preclinical obesity should be interpreted as a signal for early intervention and individualized follow-up, rather than a definitive diagnosis.

In this study, clinical criteria such as exertional dyspnea, sleep apnea, and orthopedic symptoms, were observed in only four patients, all of whom met the criteria for obesity according to different classification methods. This low prevalence may be influenced by the athletic nature of the study population, which contrasts with more sedentary pediatric cohorts. Since preclinical signs of organ dysfunction are less common in childhood due to shorter exposure to obesity-related stressors and greater physiological capacity for repair, early diagnosis remains essential. Further research is needed to refine and validate this and other classification models for use in pediatric populations, ensuring they are both clinically applicable and sensitive to the unique characteristics of childhood obesity.

Given the significant burden of pediatric obesity in Mexico (31), previous efforts to incorporate waist-to-hip ratio (WHR) and biochemical markers alongside BMI have been essential in enhancing the accuracy of obesity assessment, particularly in evaluating fat distribution and metabolic risk (18, 19). While these parameters have been widely suggested, their implementation in clinical practice has lacked a standardized, structured approach. The 2025- OCF addresses this by providing a comprehensive, stepwise diagnostic model: initiating with BMI as an initial screening tool, followed by an assessment of body fat distribution, and subsequently incorporating biochemical and clinical parameters to refine diagnosis (6). This structured approach allows for a more precise and clinically relevant evaluation, overcoming the limitations of BMI alone. Adopting this refined classification system could enhance prevention and treatment strategies, ensuring that clinical decisions are based on a child's true metabolic and physiological profile rather than BMI alone. Future research should focus on validating this approach in broader and more diverse pediatric populations, as well as developing scalable strategies for its effective integration into routine clinical practice.

A more individualized approach to obesity classification has the potential to transform pediatric obesity care, shifting from a static weight-based model to a dynamic, risk-centered framework. This transition could improve long-term metabolic outcomes, optimize early intervention strategies, and ultimately enhance clinical decision-making for children at risk.

## 5 Conclusion

This study identified significant discrepancies between BMI-based classification, body fat percentage, and the 2025-OCF in a pediatric population of young athletes. BMI misclassified several children with high skeletal muscle mass as obese, while the 2025- OCF identified a substantial number of cases of preclinical obesity that BMI alone failed to detect. These findings highlight the need for a more precise classification system that differentiates adiposity from lean mass while incorporating metabolic risk markers. Expanding this analysis to non-athletic pediatric populations will be essential to assess its broader applicability.

Beyond classification models, selecting practical and accessible body composition assessment tools is crucial for optimizing health monitoring and intervention strategies. While BIA offers a more feasible alternative to DXA, its availability remains limited in certain healthcare settings. In contrast, WHR has been extensively studied and is a widely accessible, low-cost anthropometric measure that correlates with visceral adiposity and metabolic risk, making it a viable option for large-scale implementation. The 2025-OCF provides a structured approach to obesity classification, but further validation in diverse pediatric populations is needed to refine its applicability in childhood and adolescence. Future research should focus on optimizing this model for pediatric use, ensuring its effectiveness in different clinical settings. Once validated, its integration into clinical protocols and pediatric health guidelines could enhance obesity risk stratification, promote early intervention, and improve long-term metabolic health outcomes.

## Data Availability

The raw data supporting the conclusions of this article will be made available by the authors, without undue reservation.
